# 

**Published:** 1914-01

**Authors:** 


					﻿PUBLISHED MONTHLY.	ATLANTA	SUBSCRIPTION $1.00
JOURNAL-RECQHEFf
OF MEDICINES!
i Atlanta Medical and Surgical Journal, Established 1855. ) rah
and Southern Medical Record, Established 1870.	) DUlll I CAT
Vol. LX.	Atlanta, Ga., January, 1914.	No. 10
				

